# Exploratory analysis of pre and postoperative risk stratification tools to identify acute kidney and myocardial injury in patients undergoing surgery for chronic subdural haematoma

**DOI:** 10.1097/ANA.0000000000000796

**Published:** 2021-08-23

**Authors:** Daniel James Stubbs, Benjamin M Davies, Rowan Burnstein, Alexis J Joannides, Ari Ercole

**Affiliations:** 1 Wellcome Trust Research Fellow & Honorary Specialist Registrar, University of Cambridge, Division of Anaesthesia; 2 Clinical Research Fellow & Specialist Registrar, Department of Clinical Neurosciences, University of Cambridge; 3 Consultant, University of Cambridge, Division of Anaesthesia; 4 NIHR Brain Injury MedTech Co-operative, Department of Clinical Neurosciences, University of Cambridge

## To the Editor

Perioperative statistical risk stratification is widespread. Such tools inform intraoperative and postoperative care as part of the National Emergency Laparotomy Audit (NELA)^1^.

Patients with chronic subdural haematomas (cSDH) are often elderly with significant comorbidity^2^. Despite this, there is a paucity of literature pertaining to risk stratification models in this cohort^3^. At our centre, as part of a multidisciplinary improvement initiative (the ‘Improving Care in Elderly Neurosurgery Initiative’ (ICENI)^4^) (Project ID:PRN7705) we demonstrated a significant association between postoperative complications and length of stay^2^. As a further analysis within this cohort of operated cSDH, we explore the potential of using retrospective electronic health record (EHR) data to generate prognostic statistical models for the identification of two end-organ complications (myocardial injury –troponin above the upper limit of normal and acute kidney injury (AKI) –a rise in serum creatinine of ≥ 1.5 times baseline). Outcomes were chosen based on data availability and veracity as well as clinical relevance. The integrated nature of our EHR permitted incorporation of variables reflecting intraoperative management. This enabled an exploratory analysis of models that, analogous to NELA, could be used preoperatively and updated postoperatively.

Logistic regression models were built using variables available prior to (age, American society of Anesthesiologists (ASA) score, creatinine, antithrombotic use, inter-hospital transfer, pre-operative physiological state, and comorbidities), and end of (opioid dose, length of wait, time with mean arterial pressure, (MAP) <80mmHg, time with end tidal carbon dioxide (ETCO_2_) outside of 3-5kPa, and volatile *v* intravenous anaesthetic maintenance), surgery. Physiological state was encapsulated on each admission day using the electronic postoperative morbidity score (ePOMS)^5^(details in supplemental digital content). Full details of variable generation are published elsewhere^2^. Missing data was handled by multiple imputation^6^. This was used in two ways. Firstly, *m=40* imputed datasets were formed to permit univariable screening (carrying forward all with *p* <0.2) and sequential simplification of the multivariable model using pooled likelihood ratio tests (LRT). These models were subsequently internally validated using *k*-fold (*k*=10) cross-validation using a ‘fold then impute’ strategy to minimise bias^7^. Model building and LRT results are in Supplemental Digital Content. All analysis was conducted in R v3.5.3^8^.

This study utilised a previously identified, retrospective cohort of 531 consecutive cases of primary operation for cSDH between October 2014 and January 2019, with appropriate outcome data^2^. 53 individuals suffered myocardial injury, 24 AKI. 69 had at least one ‘end-organ’ complication. After multivariable model building (*See* Supplemental Digital Content Figure S2) an admission model containing ASA, an indicator of tertiary transfer, anti-thrombotic use, and admissions ePOMS score was formed (**Model 1** in Table 1). These were supplemented with significant day of surgery variables and the process repeated. The resulting model contained ASA, tertiary transfer, anti-thrombotic use, day of surgery ePOMS, intraoperative fentanyl dose, and time out of ETCO_2_ range (**Model 2** in Table 1). Models yielded AUCs of 0.81(SD=0.01) and 0.85 (SD=0.01) after cross-validation (Supplemental Digital Content Figures S3 and S4).

Our work, despite being a single centre study and lacking external validity, demonstrates the possibility of using routinely-collected data to generate statistical models for the identification of postoperative complications after cSDH surgery. The retrospective nature of our data and the limitations of diagnostic and operative coding in cSDH^2^ means we have not been able to include all potentially relevant explanatory variables (e.g. severity of cSDH). This is one of many challenges in developing prognostic models in cSDH. For instance, the apparent protective association for transferred patients reflects right censoring, due to the absence of complication data after discharge from our centre. Improved data linkage between centres is required to accurately generate models to predict complications in such patients.

Our pre-surgery model could be calculated in any centre as the increment in discriminatory performance in model 2, although statistically significant, is likely clinically unimportant. For example, the apparent protective association with fentanyl dose could be identifying a subset of patients, deemed able to tolerate higher doses by their anaesthetist. The increased odds seen with variation in ETCO_2_ could represent patients with low cardiac output or raised intracranial pressure (requiring hyperventilation).

Further work in larger cohorts, with appropriately linked outcome data, is required to validate our approach and build on the exploratory analysis reported here to determine clinical utility.

## Supplementary Material

Supplementary Material

## Figures and Tables

**Table 1:** Final models built from admission variables (Model 1) and after the addition of intraoperative events (Model 2) for identifying end-organ complications (myocardial injury or acute kidney injury) in a cohort of *n = 531* patients undergoing surgery for chronic subdural haematoma. AUC Model **1: 0.81(SD = 0.01), AUC Model 2: 0.85(SD=0.04)** *ASA = American Society of Anesthesiologists, AUC =* Area under the receiver operator characteristic curve, *ePOMS = Electronic postoperative morbidity score, ETCO_2_ = End tidal carbon dioxide, kPa = Kilopascals, min = minutes*

Variable	Odds Ratio [95% Confidence Interval]	*p*
**Model 1: Preoperative model**		
*ASA*	2.188 [1.351 − 3.546]	0.002
*Tertiary Transfer*	0.411 [0.174 − 0.975]	0.044
*Anti-thrombotic use*	3.143 [1.726 − 5.722]	<0.001
*Admission ePOMS* (per 1 domain increase)	1.300 [1.086 − 1.544]	0.004
**Model 2: Postoperative model**		
*ASA*	2.091 [1.242 − 3.521]	0.006
*Tertiary Transfer*	0.284 [0.115 − 0.706]	0.007
*Anti-thrombotic use*	3.626 [1.903 − 6.907]	<0.001
*Day of Surgery ePOMS* (per 1 domain increase)	1.395 [1.131 − 1.720]	0.002
*Intraoperative Fentanyl* (per 25mcg)	0.839 [0.759 − 0.926]	<0.001
*Time outside of ETCO_2_ range 3-5kPa* (per 10 min)	1.325 [1.095 − 1.603]	0.004
