# Supplementary material for: Exploratory analysis of pre and postoperative risk stratification tools to identify acute kidney and myocardial injury in patients undergoing surgery for chronic subdural haematoma
Source: J Neurosurg Anesthesiol. Author manuscript; Available in PMC 2022 Oct 1. (PMC7613591; doi:10.1097/ANA.0000000000000796)

AUTHOR ACCEPTED MANUSCRIPT
CC-BY License applied

Exploratory analysis of pre and postoperative risk stratification tools to identify acute kidney and myocardial injury in patients undergoing surgery for chronic subdural haematoma.

Stubbs DJ, Davies BM, Burnstein R, Joannides A, Ercole A

Contents:

1) **Cohort details and missing data**
2) **Calculation of an electronic postoperative morbidity score (ePOMS)**
3) **Approach to the handling of missing data**
4) **Univariable screening**
5) **Model building process (incl. excluded variables and *p* values)**
6) **R Code (github link)**

Section 1: Cohort details and missing data

Full details of patterns of missing data and the cohort's characteristics have been previously published and are available here and summarised briefly below.

Of note in our previously published study one patient had missing formal discharge data (and thus an inaccurate length of stay). In this study we had necessary laboratory results (and thus outcome data) to include them, giving us a total cohort of *n* = 531.

Variable	Median [IQR]
Age *years*	77 [69-84]
Creatinine $\mu mol/l$	73 [61-89]
	**n (%)**
Male	376 (70.8)
ASA $\geq$ 3	271 (61.0)*
Cognitively Impaired	270 (54.5)*
Admission GCS 15	342 (64.4)
Admission Motor Score 6	490 (92.3)
mRS $\geq$ 2	105 (31.0)*
Anticoagulants/Antiplatelets	233 (43.9)
CVS Disease	239 (45.0)
Heart Failure	101 (19.0)
Airways Disease	75 (14.1)

*ASA = American society of anesthesiologists score, GCS = Glasgow Coma Scale, mRS = Admission Modified Rankin Score, CVS = Cardiovascular, Motor score refers to score on the motor (movement) component of the GCS. * indicates that value is calculated only on those with recorded values (see missing data)*

Four variables had missing data;

- Baseline creatinine (*n*= 46 - 8.7%)
- Cognitive status (*n*= 36 - 6.8%),
- ASA score (*n*= 87 - 16.1%),
- mRS (*n*= 192 - 36.2%)

Patterns of missing data:

- mRS alone (*n = 133* – 25.0%)
- ASA alone (*n = 46* – 8.7%)
- Creatinine + mRS (*n = 13* – 2.4%)
- Creatinine alone (*n = 10* – 1.9%)
- Cognitive status + mRS (*n = 10* – 1.9%
- Cognitive Status alone (*n = 9* – 1.7%)
- Creatinine + ASA (*n = 6* – 1.1%)
- Cognitive status, creatinine, mRS (*n = 5* – 0.9%)
- Cognitive status + Creatinine (*n = 4* – 0.8%)
- Cognitive status + creatinine + ASA (*n = 3* – 0.6%)
- Creatinine ASA + mRS (*n = 3* – 0.6%)
- Cognitive status + ASA + mRS (*n = 3* – 0.6%)
- Cognitive status + ASA + mRS + Creatinine (*n = 2* – 0.4%)
- Cognitive status + ASA (*n = 1* – 0.2%)

CC-BY License applied

Section 2: Calculation of an electronic postoperative morbidity score (ePOMS)

Domain	Diagnostic Criteria	Notes
**Respiratory**	Need for supplementary oxygen	
**Cardiovascular**	HR >100<br>SBP <100<br>Positive Troponin test	
**Neurological**	Need for nurse special	Nurse special used as surrogate for confusion/delirium
	Motor/Verbal Score worse than referral*	Motor/Verbal Score refers to specific domains of the GCS
	Focal neurology*	Documented mismatch between left/right sided arm/leg strength at any stage
**Renal**	Rise in creatinine to ≥ 1.5x baseline	Baseline creatinine taken as last recorded creatinine prior to surgery.
**GI**	Anti-emetic administered	Anti-emetic defined by following WHO ATC codes: a04**, a03fa01, r06ae03, n05ad08
**Pain**	Need for IV opioids or local anaesthetic infusion	Drugs identified by following WHO ATC codes: n02aa01i, n02ab03i, n01bb01
**Recurrence**$^{\%}$	Reoperation	EPOMS originally identifies severe wound infection by need for further surgery. In this context reoperation for the same procedure is being used to identify re-accumulation of cSDH
**Infection**	Temperature ≥ 38°C<br>Receiving Antibiotics	Antibiotics defined by following WHO ATC codes: j01**
**Haematological**	Transfused with blood product	Including red cells, platelets, FFP, cryo-precipitate

*HR = Heart Rate, SBP = Systolic blood pressure, GCS = Glasgow Coma Scale, GI = Gastrointestinal system, WHO ATC = World Health Organisation Anatomical therapeutic chemical classification, IV = intravenous, FFP = fresh frozen plasma. If multiple potential criteria are listed then an individual scores if any of these are met % In the original EPOMS score this would correspond to the 'wound' category. * Indicates additional criterion included in this variant from previously published*[1]. *** indicates that all drugs below this level of ATC code were included*

AUTHOR ACCEPTED MANUSCRIPT
CC-BY License applied

1. Stubbs DJ, Bowen JL, Furness RC, Gilder FJ, Romero-Ortuno R, Biram R, Menon DK, Ercole A (2019) Development and Validation of an Electronic Postoperative Morbidity Score. Anesth Analg 129(4):935–942

Section 3: Approach to the handling of missing data

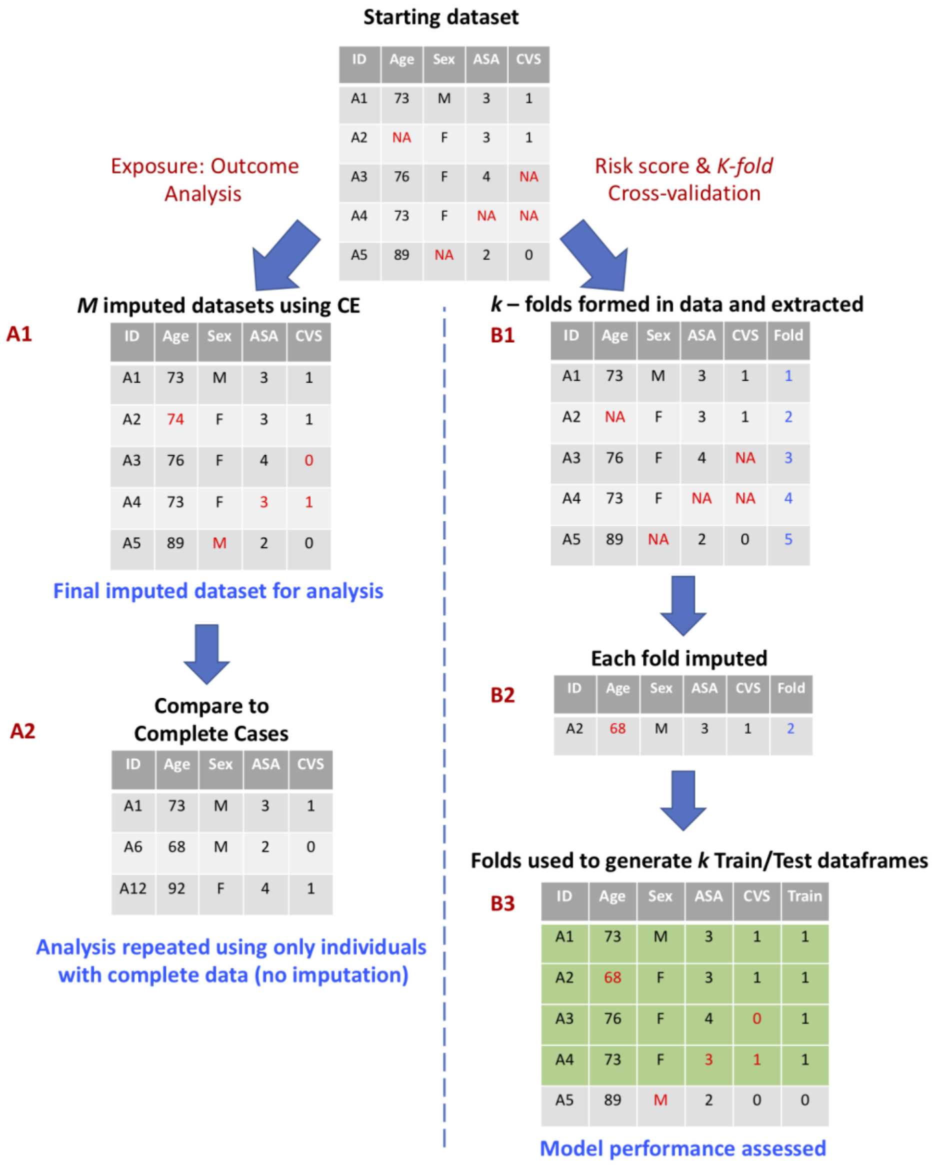

Supplementary Figure S1: Approach to the handling of missing in data in model building (A1-A2) and in combination with *k-fold* cross validation for internal validation of generated model.

For assessment between exposures (variables) and outcome of interest, missing baseline variables were imputed using `multiple imputation using chained equations' (MI with CE) (**A1**). These results were compared to complete cases results for each analysis (**A2**). A distinct approach was used to allow internal validation of final multivariable models. This was done with *k=10* fold cross-validation. The dataset was split into test/train folds (**B1**), test folds were then individually imputed (**B2**), before being recombined (**B3**). Fold indices

CC-BY License applied

created in **B1** were use d to perform cross validation forming sequential training (green rows) and test (grey row) datasets.

Section 4: Univariable screening

	OR	95% Lower	95% Upper	*p*
**Age** *per year*	1.002	0.982	1.024	0.819
**Male** *versus Female*	0.641	0.378	1.086	0.099
**ASA2** *All versus ASA 1*	0.848	0.097	7.373	0.881
**ASA3**	3.126	0.405	24.137	0.275
**ASA4**	13.260	1.612	109.067	0.017
**ASA5**	0.832	0.000	3.000	0.999
**mRS 1** *All versus mRS 0*	1.042	0.500	2.171	0.913
**mRS 2**	1.337	0.524	3.407	0.544
**mRS 3**	1.846	0.674	5.056	0.234
**mRS 4**	0.892	0.259	3.071	0.856
**Tertiary transfer** *versus direct admission*	0.340	0.161	0.720	0.005
**Cognitive Impairment**	3.005	1.640	5.506	<0.001
**M6 on admission** *versus any other motor score*	0.244	0.121	0.493	<0.001
**GCS 15 on admission** *versus any other GCS*	0.396	0.237	0.661	<0.001
**Creatinine** *per 20µmol/l*	1.127	1.020	1.269	0.031
**History of CVS Disease**	2.410	1.426	4.072	0.001
**Anticoagulant on admission**	4.359	2.468	7.699	<0.001
**Airways Disease**	2.755	1.516	5.008	0.001
**Heart Failure**	1.920	1.083	3.404	0.026
**Admission EPOMS** *per 1 domain increase*	1.513	1.290	1.775	<0.001
**DOS EPOMS** *per 1 domain increase*	1.668	1.379	2.017	<0.001
**Pre-op Deterioration**	0.613	0.367	1.023	0.062
**Length of Wait** *per hour*	1.005	1.000	1.011	0.069
**Op Time** *per 10 min*	1.062	1.001	1.127	0.057
**Volatile anaesthetic** *versus TIVA*	0.964	0.579	1.606	0.889
**Fentanyl dose** *per 25 mcg*	0.839	0.758	0.928	<0.001
**Time MAP not 80mmHg** *per 10 min*	0.961	0.886	1.041	0.346
**Time CO2 not 3-5kPa** *per 10 min*	1.293	1.116	1.509	0.001

Supplementary Table S1: Pooled univariable analysis for the identification of end-organ complications (myocardial infarction or acute kidney injury) in a cohort of 531 cases of operated chronic subdural haematoma. Analysis conducted across m = 40 multiply imputed datasets. *ASA = American Society of Anesthesiologists score, CO2 = End Tidal Carbon Dioxide tension, CVS = Cardiovascular System, DOS = Day of Surgery, EPOMS = Electronic postoperative morbidity score, GCS = Glasgow Coma Scale, kPa = kilopascals, MAP = mean arterial pressure, mcg = micrograms, min = minutes, M6 = Motor score of 6 on the Glasgow coma scale, mRS = Modified Rankin Scale, TIVA = Total Intravenous Anaesthesia, Length of wait = wait between admission and surgery.*

Section 5: Model building process and results for identification of end-organ complications:

After a process of univariable screening, all variables with *p<0.2* were carried forward to multivariable model building. This was performed by pooling results across 40 multiply imputed datasets and compared to complete cases.

CC-BY License applied

Supplementary Figure S2 demonstrates model building process. After univariable testing a starting model formed from variables available to clinicians **prior to surgery** and the subsequent order of exclusion of variables using backwards step regression with a threshold for exclusion of $p=0.05$ on the pooled likelihood ratio test.

This final `pre-op model' **(Model 1 in Table 1)** was then further refined by the addition of information available at the conclusion of surgery (DOS = Day of Surgery variables) with an equivalent process of model refinement using backwards step regression. This resulted in a final `post-op model' (**Model 2 in Table 2).**

Both models were subsequently tested using internal validation with a distinct imputation method (see **Supplementary Figure S1**) and discrimination assessed using the area under the receiver operator characteristic curve (ROC) (**Supplementary Material Figures S3 and S4)**

Section 6: R Code

Although data for this cannot be made available due to its potentially sensitive nature and origins within an approved service evaluation project we have made our analytical code available on GitHub here.

AUTHOR ACCEPTED MANUSCRIPT
CC-BY License applied

Supplementary Figure S2:
Summary of Multivariable model building

Starting model:

	OR	95% Lower	95% Upper	p
**Male**	0.610	0.331	1.119	0.110
**ASA**	1.670	0.953	2.926	0.073
**M6 on Admission**	0.720	0.276	1.882	0.502
**GCS15 on Admission**	0.747	0.388	1.439	0.383
**Cognitive Impairment**	1.727	0.810	3.682	0.157
**CVS Disease**	1.843	0.929	3.657	0.080
**Creatinine**	1.127	0.961	1.321	0.090
**Airways Disease**	1.580	0.758	3.290	0.221
**Heart Failure**	0.769	0.359	1.645	0.497
**Anticoagulant**	2.867	1.526	5.384	0.001
**Tertiary Transfer**	0.455	0.168	1.230	0.120
**Admission ePOMS**	1.256	1.048	1.506	0.014

Sequential removal of variables based on *p*

Variable removed	*P prior to removal*	*P (LRT to previous)**
M6 on admission	0.502	0.501
Heart Failure	0.519	0.519
GCS15	0.222	0.195
Airways Disease	0.196	0.206
Male	0.114	0.144
Creatinine	0.130	0.150
Cognitive Concern	0.086	0.074
CVS Disease	0.069	0.066

ASA, Tertiary Transfer, Anticoagulation, Admission ePOMS carried forward

Add in DOS variables:

	OR	95% Lower	95% Upper	p
**ASA**	1.890	1.107	3.225	0.022
**Tertiary Transfer**	0.304	0.116	0.796	0.015
**Anticoagulant Use**	3.450	1.793	6.637	<0.001
**Admission ePOMS**	1.200	0.984	1.458	0.071
**DOS ePOMS**	1.300	1.035	1.630	0.024
**Operation time (per 10 min)**	1.020	0.944	1.104	0.603
**Fentanyl dose (per 25mcg)**	0.840	0.764	0.940	0.002
**Time MAP <80mmHg (per 10 min)**	0.949	0.856	1.053	0.325
**Time outside of $ETCO_2$ 3-5kPa (per 10 min)**	1.308	1.072	1.594	0.008
**Length of Wait (per hr)**	1.005	0.999	1.011	0.120

Sequential removal of variables based on *p*

Variable removed	*P prior to removal*	*P (LRT to previous)**
Operation time	0.603	0.599
Time MAP <80mmHg	0.399	0.388
Length of Wait	0.126	0.129
Admission ePOMS	0.120	0.116

Final model: ASA, Anticoagulant use, DOS ePOMS, Fentanyl dose, Time out of CO2 range, Tertiary Transfer.

AUTHOR ACCEPTED MANUSCRIPT
CC-BY License applied

Supplementary Figure S3: Receiver Operator Characteristic (ROC) curve for model using admission variables

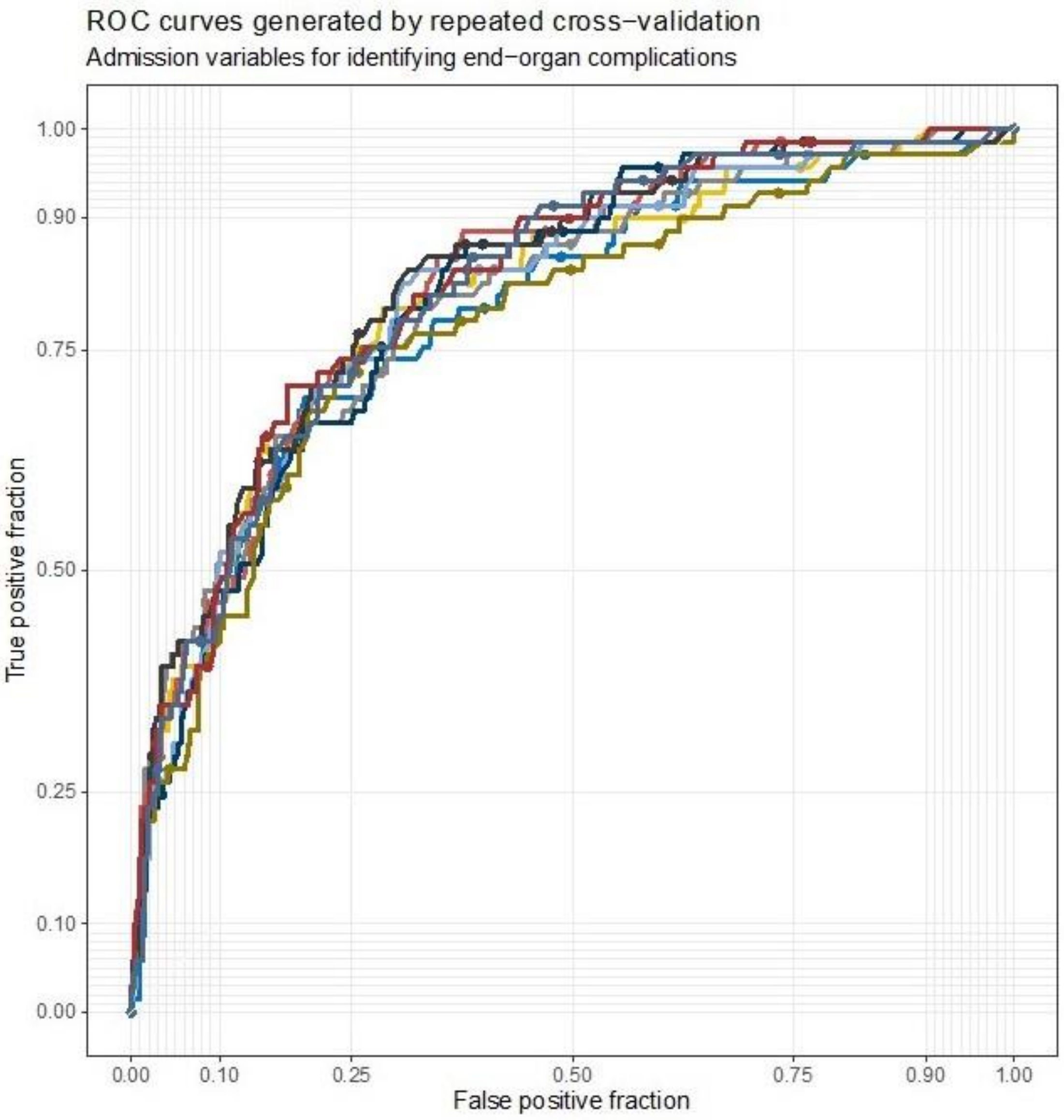

AUTHOR ACCEPTED MANUSCRIPT
CC-BY License applied

Supplementary Figure S4: Receiver Operator Characteristic (ROC) curve for model using day-of-surgery variables

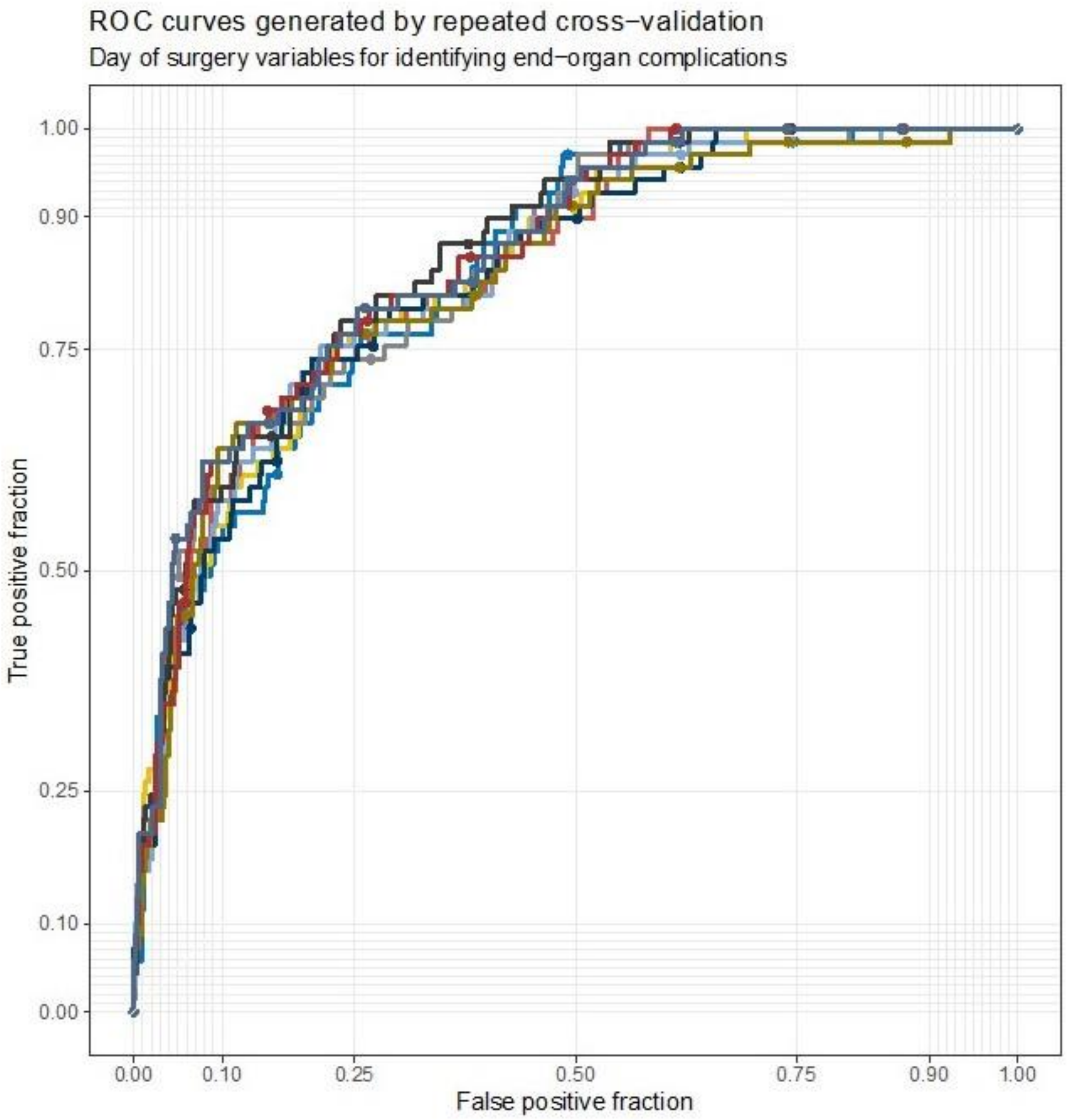

Supplement: Supplementary Material [file EMS132502-supplement-Supplementary_Material.pdf]
